# Does social support moderate the association between hunger and mental health in youth? A gender-specific investigation from the Canadian Health Behaviour in School-aged Children study

**DOI:** 10.1186/s12937-020-00648-3

**Published:** 2020-12-05

**Authors:** Nour Hammami, Scott T. Leatherdale, Frank J. Elgar

**Affiliations:** 1 Institute for Health and Social Policy, McGill University, 1130 Pine Avenue West, Room B4, Montreal, Quebec H3A1A3 Canada; 2 School of Public Health and Health Systems, University of Waterloo, 200 University Avenue West, Waterloo, ON N2L3G1 Canada; 3 Institute for Health and Social Policy and Department of Psychiatry, McGill University, 1033 Avenue des Pins, Montreal, Quebec H3A1A1 Canada

**Keywords:** Social support, HBSC, Canada, Youth, Adolescents, Mental well-being, Mental health, Hunger, Gender

## Abstract

**Background:**

Youth who go hungry have poorer mental health than their counterparts – there are gender differences in this relationship. This study investigated the role of social support in the association between hunger and mental health among a nationally representative sample of youth in Canada in gender-specific analyses.

**Methods:**

We used a probability-based sample of 21,750 youth in grades 6–10 who participated in the 2017–2018 Canadian Health Behaviour in School-aged Children. Self-report data were gathered on hunger, mental health (measured via the World Health Organization-5 well-being index) and five sources of support – peer, family and teacher support as well as the school climate and neighborhood support. We conducted adjusted, gender-specific, multilevel regression analyses assessing the association between mental health, social support and hunger.

**Results:**

We found that youth who reported lower support were more likely to experience going to bed hungry (relative to never hungry) across all support factors. As for the social support factors, all the social support factors were associated with a higher mental health score, even after controlling for hunger. Despite these results our final set of models showed that our measures of social support did not alleviate the negative association between hunger and mental health. As for gender-specific findings, the negative association between hunger and a mental health was more pronounced among females relative to their male counterparts. We also found that certain social support factors (i.e., family, teacher and neighborhood support) were associated with a higher mental health score among females relative to males while controlling for hunger status.

**Conclusions:**

We find that five social support factors are associated with a higher mental health score among ever hungry youth; however, social support did not overpower the negative association between hunger and mental health. Food insecurity is a challenge to address holistically; however, hungry youth who have high social support have higher odds of better mental health.

**Supplementary Information:**

The online version contains supplementary material available at 10.1186/s12937-020-00648-3.

## Background

Research and policy agendas are increasingly directing their attention to malnourishment, with special attention directed towards the globally high and increasing rates of overweight and obesity. However, on the other end of the spectrum: the global prevalence of undernourishment, by being food insecure every day, is estimated at 2 billion individuals (equivalent to 26.4% of the world’s population) [1]. Food insecurity is commonly defined as the “inability to obtain sufficient, nutritious, personally acceptable food through normal food channels or the uncertainty that one will be able to do so” [2]. The most recent findings (2017/2018) indicated that the prevalence of household food insecurity was estimated at 12.7% in Canada [3].

Of specific concern in Canada, households with children were found to be at a greater disadvantage of being food insecure than households without children. Among households consisting of couples without children, 3.4% were food insecure; while, across households consisting of couples with children (less than 18 years old) the number more than doubles: 7.3% had food insecurity [3]. This is problematic for those who have restricted their eating and are hungry (i.e., have severe food insecurity) [4].

Among youth, hunger is associated with reduced academic performance, risky behaviours (e.g., substance use) and poor mental health [5–7]. The Public Health Agency of Canada defines mental health as “the capacity of each and all of us to feel, think, and act in ways that enhance our ability to enjoy life and deal with the challenges we face. It is a positive sense of emotional and spiritual well-being that respects the importance of culture, equity, social justice, interconnections and personal dignity” [8]. Among youth in Canada, hunger was found to be associated with poor mental health – even after controlling for socio-economic status [9]. Furthermore, the association between hunger and poor health may be stronger in female youth compared to males [10]. Hunger’s relationship with poor mental health persists after controlling for socio-economic status and is different across the genders; however, there is controversy on how and why hunger contributes to poorer mental health. This may be partly due to the lack of evidence on protective factors for youth that report hunger. If there are associations with psychosocial factors, then environmental moderators of stress are candidates for moderators of the hunger-mental health association.

The social ecological model suggests that it is important to understand the relationship between interpersonal (i.e., social factors) and health correlates (e.g., hunger) and their association with mental health. Previous research by Pickett et al. [9] investigating how individual level factors play a role in the association between hunger and mental health among youth in Canada supports this premise. Additionally, social support is a factor that is associated with positive mental health [11]. As for its association with stress, social support moderates stress pathways [12] and could moderate the link to hunger. The general benefits model suggests that social support is positively associated with positive health effects and negatively associated with negative health effects [13]. Research that followed this seminal article found that not all social support factors are associated with positive health effects; rather, these effects may differ depending on the type of support being studied [14]. Therefore, it is important to understand a variety of social support factor’s roles (i.e., social support as a moderator) in the association between hunger and mental health.

One global and two population-specific studies investigated social support as a moderator for hunger and mental health among adult populations, with mixed findings. One study used Gallup World Poll (GWP) data from 138 countries and reported that there was no evidence that social support (positively or negatively) moderated the relationship between food insecurity and mental health [15]. Another study from the GWP found that social support positively moderated the association between hunger and mental health in sub-Saharan Africa [16]. A third study assessed for the moderating role of social support on food insecurity and depression among Latin Americans living with type 2 diabetes; the authors reported that the risk of depression was lower at each level of food insecurity when social support was high [17]. With these mixed findings among adults and no identified reports on this matter among youth in Canada, it would be informative to examine the role of social support as a moderator between mental health and hunger among youth in Canada.

Youth are exposed to social influences from different groups. Whether social support mitigates the negative association between hunger and mental health has not been closely investigated among youth. Furthermore, stress (e.g., hunger) reportedly has a stronger effect on female youth’s mental health (i.e., depression or anxiety) relative to their male counterparts [18]; therefore, a gender-specific investigation is warranted. As such, the aim of this study was to investigate the role of social support from peers, family members, teachers, schools and neighborhoods in the association between hunger and mental health among youth across Canada in gender-specific analyses.

## Methods

### Data source: Health Behaviour In School-Aged Children Study (HBSC)

The HBSC - World Health Organization collaborative study is a cross-national initiative across countries in Europe and North America. It aims to assess youth’s health behaviours, social environment and outcomes by having youth fill surveys on their physical, social and emotional health. The school-based survey is conducted during youth’s normal classroom setting and takes approximately 45 min. Additional details on the HBSC study and design are available elsewhere [19, 20]. Ethics approval was granted by the General Research Ethics Board at Queen’s University, the Public Health Agency of Canada and Health Canada’s Research Ethics Board.

This study used data provided by youth in Canada in the 2017/2018 survey cycle. These constituted 21,750 youth in grades 6 through 10 from 287 schools across 12 Canadian provinces and territories. HBSC-Canada consists of a probability-based sample of youth (i.e., nationally representative) using both active- information with active-consent and passive-consent approaches depending on school board requirements. The mean student participation rate from participating classes is 75%.

### Measures

#### Mental health

Mental health was measured via the World Health Organization-5 (WHO-5) mental well-being index which is valid for use among youth when measuring emotional functioning and screening for depression [21]. The WHO-5 well-being index consists of a score based on youth’s responses to five questions related to their positive mood, vitality and general interest during the past 2 weeks: “I have felt cheerful and in good spirits”, “I have felt calm and relaxed”, “I have felt active and vigorous”, “I woke up feeling fresh and rested” and “My family life has been filled with things that interest me” – rated on a six item scale of 1 (at no time) to 6 (all of the time). For this analysis, the summed WHO-5 mental well-being index raw scores (ranging from 5 to 30) were transformed to an ordinal variable based on tertial groups: low, middle or high.

#### Hunger

Hunger was assessed by the question “Some young people go to school or to bed hungry because there is not enough food at home. How often does this happen to you?”. Four options were available: always, often, sometimes and never. Since fewer than 4% of youth identified as “always” or “often” hungry, we combined these categories with the “sometimes” hungry youth and named them “ever hungry” youth versus “never hungry”.

#### Social support

Five measures of social support were included in this study: friend, family, teacher, school climate and neighborhood – they are detailed below.

##### Friend support

Friend support was measured as a score from four questions regarding the extent to which: their friends try to help them, they can count on them, they can share with them happy and sad feelings and they can talk to them about their problems – rated on a seven item scale of 1 (very strongly disagree) to 7 (very strongly agree) (Cronbach’s alpha: 0.937). The summed scores (ranging from 4 to 28) were transformed to an ordinal variable based on tertial groups: low, middle or high.

##### Family support

Family support was measured from four questions regarding the extent to which: their family tries to help them, they receive support from them, they can talk with them about their problems and their willingness to help them make decisions – rated on a seven item scale of 1 (very strongly disagree) to 7 (very strongly agree) (Cronbach’s alpha: 0.926). The summed scores (ranging from 4 to 28) were transformed to an ordinal variable based on tertial groups: low, middle or high.

##### Teacher support

Teacher support was measured from eight questions regarding the extent to which teachers: accept them, care about them, can be trusted, are available if students need extra help, are interested in them, are friendly, encourage them to express their views and treat them fairly – rated on a five item scale of 1 (strongly agree) to 5 (strongly disagree) (Cronbach’s alpha: 0.898). The summed scores (ranging from 8 to 40) were transformed to an ordinal variable based on tertial groups: low, middle or high.

##### Supportive school climate

Supportive school climate was measured from four questions regarding the extent to which the school: has fair rules, is a nice place to be, they feel they belong and the extent to which they like the school – rated on a five item scale of 1 (strongly agree) to 5 (strongly disagree) (Cronbach’s alpha: 0.811). The summed scores (ranging from 4 to 20) were transformed to an ordinal variable based on tertial groups in the sample: low, middle or high.

##### Neighborhood support

Neighborhood support was measured from five factors, the extent to which youth feel that: people say hello and talk to each other, safety of young children to play, they can trust people, there are good places where they can spend their time and they could ask for help or favours from neighbours – rated on a five item scale of 1 (strongly agree) to 5 (strongly disagree) (Cronbach’s alpha: 0.783). The summed scores (ranging from 5 to 25) were transformed to an ordinal variable based on tertial groups: low, middle or high.

### Control variables

The control variables included: school-grade, ethnicity, urban status and socio-economic status (SES). Urban status referred to the status of the municipality: rural, small, medium or large urban. Ethnicity was self-identified via the question: “People living in Canada come from many different cultural and racial backgrounds. How do you describe yourself?”. There were 16 options for youth to choose from. They were grouped into the eight most reported options: White, Black, Latin American, Indigenous (First Nations, Métis or Inuit), East and Southeast Asia (e.g., Cambodian, Indonesian), East Indian and South Asian (e.g., East Indian, Pakistani), Arab and West Asian (e.g., Afghan) and Other (including mixed ethnicities).

SES was measured using the HBSC Family Affluence Scale [22]. This scale accounts for the material resources that a family has in lieu of the traditional approach of youth reporting parental occupation and educational status; reportedly, such questions previously resulted in a large number (20–45%) of non-response values [22]. The scale consisted of six questions: “Do you have your own bedroom for yourself?”, “How many bathrooms are in your home?”, “Does your family own a car, van or truck?”, “How many times did you and your family travel out of Canada for a holiday last year?” , “Does your family have a dishwasher at home?” and “How many computers does your family own?”. The summed scores (ranging from 0 to 13) were transformed to an ordinal variable based on quintile groups: 1 (lowest quintile), 2, 3, 4 or 5 (highest).

### Statistical analyses

Summary statistics are presented stratified by gender and hunger status. We also conducted chi^2^ tests to assess for significant differences in the variables of interest across youth who are ever hungry versus never hungry within the same gender group. Multivariate analyses consisted of three sets of gender-stratified, ordinal, multilevel, regression models; all models controlled for school-grade, ethnicity, SES and urban status and adjusted for the clustered nature of the data (students within schools). The first set of models individually regressed hunger on the support factors (low versus middle/high). The second set of models, regressed mental health (low versus middle/high) onto hunger status and each of the support factors to identify whether there were associations between mental health, hunger and each support factor. Additionally, one model from this set of models regressed mental health onto hunger status and all the support factors. A third set of models regressed mental health (low versus middle/high) onto an interaction term between hunger status and the support factors (individually), to assess whether social support moderates the association between hunger and mental health. To account for variations in sampling between provinces and territories, standardized weights were used analysis via the svy toolkit. All analyses were conducted in Stata 16.0 [23] and the level of significance was set at *p* < 0.05.

## Results

### General population characteristics from youth in Canada

Table 1 presents summary statistics regarding the prevalence of hunger, mental health scores and social support across hunger status among female and male youth in Canada. The prevalence of youth with low and middle mental health was similar (low: 36.4%, middle: 36.1%); but, was higher than youth reporting high mental health (27.5%) (data not shown). This sample consisted of slightly more females than males (52.4% versus 47.6%). As for gender differences in hunger status, youth who were ever hungry were similar across the genders: 16.5 and 17.1% among females and males, respectively.

**Table 1:** Weighted summary statistics [%(n)] for youth in Canada’s general characteristics stratifying for gender and hunger status

	Females [%(n)]			Males [%(n)]		
	Ever hungry	Never hungry	Chi-2 *p*-value	Ever hungry	Never hungry	Chi-2 *p*-value
Hunger	16.5 (1829)	83.5 (9259)		17.1 (1722)	82.9 (8338)	
Mental health						
Low	22.5 (1015)	77.5 (3502)	254.1 *p* < 0.0001	22.7 (594)	77.3 (2019)	137 *p* < 0.0001
Middle	13.2 (487)	86.8 (3182)		17.9 (632)	82.1 (2894)	
High	8.3 (191)	91.7 (2114)		11.3 (365)	88.7 (2840)	
Grade						
6	17.1 (335)	82.9 (1622)	22.7 *P* = 0.2466	19.5 (383)	80.5 (1578)	14.4 *P* = 0.3858
7	19.0 (440)	81.0 (1877)		17.7 (378)	82.3 (1764)	
8	14.7 (340)	85.3 (1974)		17.0 (354)	83.0 (1731)	
9	14.5 (357)	85.5 (2103)		15.9 (350)	84.1 (1853)	
10	16.8 (324)	83.2 (1610)		15.5 (244)	84.5 (1336)	
Social support factors						
Friend support						
Low	20.1 (615)	79.9 (2445)	48.2 *P* < 0.0001	19.2 (636)	80.8 (2675)	24.8 *P* < 0.01
Middle	15.0 (565)	85.0 (3192)		16.8 (642)	83.2 (3193)	
High	16.2 (1698)	83.8 (3149)		14.1 (305)	85.9 (1860)	
Family support						
Low	24.8 (918)	75.2 (2789)	353.7 *P* < 0.0001	24.9 (682)	75.1 (2054)	217.7 *P* < 0.0001
Middle	14.7 (469)	85.3 (2732)		16.8 (538)	83.2 (2661)	
High	8.5 (298)	91.5 (3229)		10.8 (370)	89.2 (3042)	
Teacher support						
Low	24.8 (860)	75.2 (2610)	299.0 *P* < 0.0001	21.9 (616)	78.1 (2195)	87.1 *P* < 0.0001
Middle	14.6 (494)	85.4 (2886)		15.9 (520)	84.1 (2745)	
High	9.6 (348)	90.4 (3275)		13.2 (437)	86.8 (2885)	
School climate						
Low	23.0 (878)	77.0 (2939)	209.5 *P* < 0.0001	21.9 (711)	78.1 (2533)	98.9 *P* < 0.0001
Middle	14.2 (518)	85.8 (3126)		16.1 (525)	83.9 (2736)	
High	10.6 (336)	89.7 (2833)		12.7 (387)	87.3 (2664)	
Neighborhood support						
Low	22.3 (797)	77.7 (2775)	250.2 *P* < 0.0001	21.0 (600)	79.0 (2259)	85.0 *P* < 0.0001
Middle	14.9 (504)	85.1 (2879)		17.2 (524)	82.8 (2518)	
High	7.8 (228)	92.1 (2678)		11.9 (322)	88.1 (2392)	

There were significant differences in mental health across gender and hunger status. Among females and males scoring low on mental health: 22.5 and 22.7% reported being ever hungry, respectively. Ever hungry male youth reported more middle (Males: 17.9%, Females: 13.2%) and high mental health scores (Males: 11.3%, Females: 8.3%) than females. The accompanying chi-square results indicate that the mental health index scores are statistically different across hunger status among females and males.

### Hunger and social support

Table 2 presents adjusted odds ratios (OR) from the gender-specific regression models that regressed hunger (dependent variable) on the social support factors (independent variable) among female (Models 1–5) and male (Models 6–10) youth in Canada. Females and males who reported middle or high support (relative to low support) were less likely to report being ever hungry across all the support factors, while holding other variables constant (Table 2, Models 1–10) (non-significance was reported for males with middle friend support and males with middle neighborhood support). High family support was the support factor that was least associated with reporting being ever hungry among both female and male youth (Females: Odds Ratio [OR] = 0.299, 95% Confidence Intervals [C.I.] = 0.220, 0.407; Males: OR = 0.328, 95% C.I. = 0.264, 0.406), while holding other variables constant (Table 2, Models 2 and 7, respectively).

**Table 2:** Results of the binary regression models that regressed hunger status on social support factors (low versus medium/high) and control variables, among youth in Canada in gender-specific analyses. The point estimates are adjusted odds ratios and the intervals show 95% confidence intervals

	Friend support	Family support	Teacher support	School Climate	Neighborhood support
	Females				
	Model 1	Model 2	Model 3	Model 4	Model 5
Support					
Low (Reference)					
Middle	0.783**	0.566***	0.542***	0.569***	0.641***
	(0.653–0.938)	(0.456–0.703)	(0.427–0.687)	(0.461–0.703)	(0.512–0.803)
High	0.752**	0.299***	0.320***	0.384***	0.338***
	(0.630–0.897)	(0.220–0.407)	(0.251–0.410)	(0.298–0.493)	(0.263–0.434)
	Males				
	Model 6	Model 7	Model 8	Model 9	Model 10
Support					
Low (Reference)					
Middle	0.867	0.571***	0.659***	0.679***	0.830
	(0.700–1.075)	(0.467–0.697)	(0.527–0.824)	(0.549–0.839)	(0.668–1.031)
High	0.752*	0.328***	0.524***	0.500***	0.566***
	(0.587–0.963)	(0.264–0.406)	(0.424–0.647)	(0.405–0.617)	(0.454–0.705)

*** *p* < 0.001, ** *p* < 0.01, * *p* < 0.05

Control variables (not shown): grade, race, socioeconomic status, urban status

### Mental health, hunger and social support

**Fig. 1:**
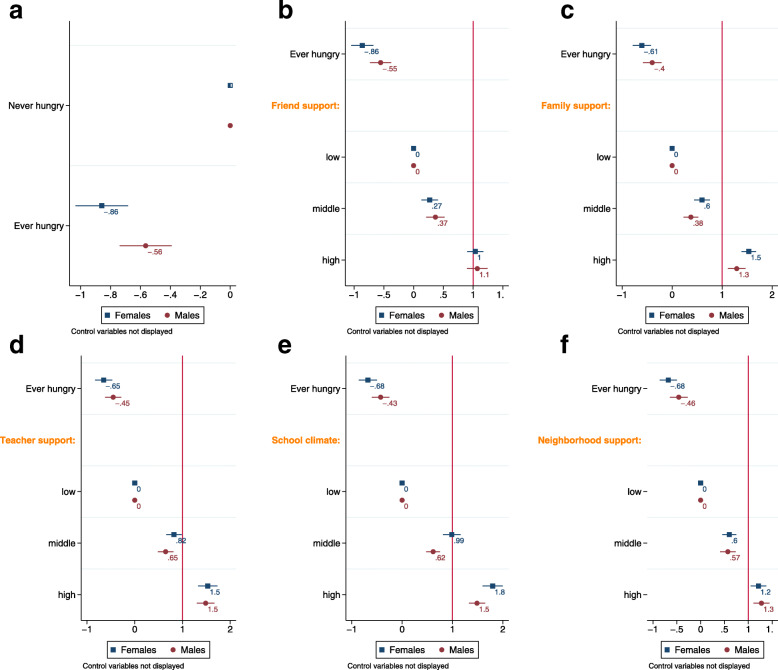
Results of the ordinal regression models that regressed mental health (low versus medium/high) on hunger, social support factors and control variables, among youth in Canada in gender-specific analyses. The point estimates are adjusted odds ratios and the error bars show 95% confidence intervals. (**a**) Hunger (**b**) Hunger and friend support (**c**) Hunger and family support (**d**) Hunger and teacher support (**e**) Hunger and school climate (**f**) Hunger and neighborhood support

Figure 1 presents a visual of the adjusted OR from the gender-specific regression models that regressed mental health (dependent variable) on hunger, the individual social support factors (independent variables), and adjusted for control variables, among female and male youth (model results are available in Supplemental Table 1). Similar to the findings from the models in Table 2, youth who were ever hungry had lower odds of middle or high support relative to never hungry youth. Furthermore, in these models all of the support factors were statistically significant and positively associated with mental health while holding other variables constant. Consistently in these models, hunger has lower odds of middle/high mental health among females than among males, suggesting that hunger is associated with poor mental health more strongly among females than males.

**Fig. 2:**
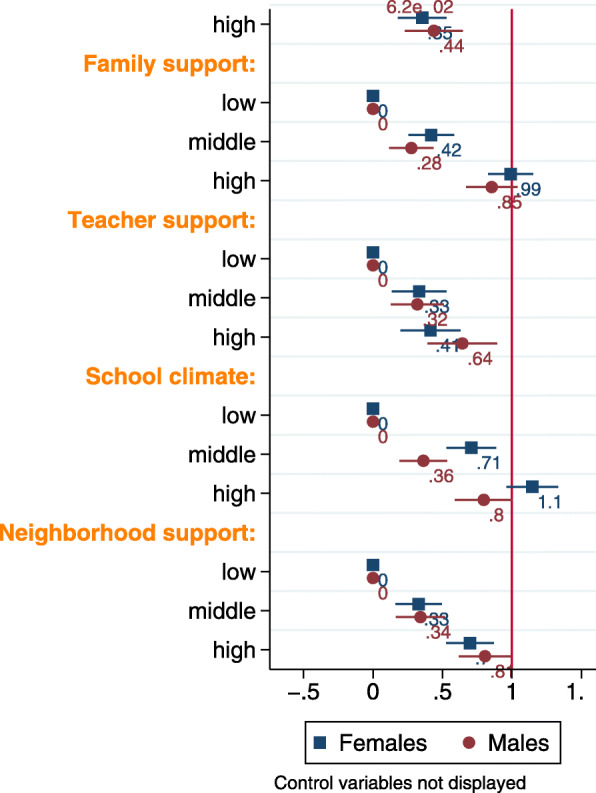
Results of the ordinal regression models that regressed mental health (low versus medium/high) on hunger, all the social support factors and control variables, among youth in Canada in gender-specific analyses. The point estimates are adjusted odds ratios and the error bars show 95% confidence intervals

Figure 2 is a visual representation of the female- and male-specific models that assessed for the association between mental health (dependent variable), hunger and all the social support factors (independent variables) while adjusting for the control variables among female and male youth (Model 7 and Model 14, respectively, model results are available in Supplemental Table 1). Ever hungry youth were associated with lower odds of middle/high mental health, relative to their never hungry peers and all the social support were positively associated with mental health (except for medium friend support), while holding other variables constant.

### Social support does not moderate hunger’s association with mental health

Table 3 presents the adjusted OR from the gender-specific regression models that assessed for the association between the mental health (dependent variable) and an interaction term between hunger and social support factors (independent factors) among female and male youth in Canada: friend support, family support, teacher support, school climate and neighborhood support.

**Table 3:** Results of the ordinal regression models that regressed mental health (dependent variable) on the interaction between social factors and hunger (independent variables) in gender-specific regression models controlling for grade, family affluence scale and urban status among youth in Canada in gender-specific analyses. The point estimates are adjusted odds ratios and the error bars show 95% confidence intervals

	Friend support	Family support	Teacher support	School climate	Neighborhood support
	Model 1	Model 2	Model 3	Model 4	Model 5
Females					
Hunger by support	–	–	–	–	–
Ever hungry at medium support	0.951	1.106	1.391	1.290	0.968
	(0.675–1.340)	(0.769–1.591)	(0.960–2.016)	(0.907–1.834)	(0.682–1.373)
Ever hungry at high support	0.581*	0.881	1.148	0.796	0.996
	(0.378–0.895)	(0.567–1.369)	(0.728–1.811)	(0.531–1.194)	(0.653–1.518)
	**Model 6**	**Model 7**	**Model 8**	**Model 9**	**Model 10.**
Males					
Hunger by support	–	–	–	–	–
Ever hungry at medium support	0.951	1.106	1.391	1.290	0.968
	(0.675–1.340)	(0.769–1.591)	(0.960–2.016)	(0.907–1.834)	(0.682–1.373)
Ever hungry at high support	0.581*	0.881	1.148	0.796	0.996
	(0.378–0.895)	(0.567–1.369)	(0.728–1.811)	(0.531–1.194)	(0.653–1.518)

*** *p* < 0.001, ** *p* < 0.01, * *p* < 0.05

Control variables (not shown): hunger status, the respective social support factor corresponding with the interaction term, grade, race, socioeconomic status, urban status

These models (the third set) did not find the interaction term to be significant. The lack of significance of the interaction term indicates the social support factors do not (positively or negatively) moderate the association between hunger and mental health.

## Discussion

Our study is a unique investigation of the role that social support plays in the association between hunger and mental health among youth in Canada in a gender-specific investigation. Approximately 1 in 6 youth in Canada reported being ever hungry, and those youth reported lower perceived social support relative to their never hungry peers. As for associations with mental health, all social support factors were associated with higher odds of better mental health while hunger was associated with lower odds of mental health. The association between hunger and poor mental health was more pronounced among female youth relative to their male counterparts. We also found that certain social support factors were associated with a higher mental health score among females relative to males. These findings indicate that female youth may respond to stress and protective factors more strongly than males. Although this is the case, perceived social support did not (positively or negatively) moderate the association between hunger and mental health among female nor male youth in Canada. Therefore, social support acts as a protective factor for mental health in adolescence; however, it does not overpower the negative association between hunger and mental health.

Previous research also supports our findings. A study among youth in economically distressed neighborhoods across five cities in different countries found that social support, via a caring female adult in the home, was positively associated with hope and negatively associated with depression among adolescents [24]. As for individuals who reported having hunger, similar to our findings, a study among women in Toronto, Ontario (Canada), also found that women who have hunger reported more isolation and less social support (V. S [25].). Another study from Canada (Quebec City, Quebec) reported that households with food insecurity were characterized by alienation and feeling like they were excluded from society since they were unable to provide for their household the proper food their family members required [26]. A common characteristic to these studies, as well as ours, is that they are conducted on a Canadian population. With food insecurity in Canada being reported at 12.7% [3], Canada is considered to have a lower relative prevalence, as some regions report a food insecurity prevalence of 62.7% (Eastern Africa) [1]. A recent global investigation found that mental health complaints were more common in areas where food insecurity is less common and more stigmatized as reported from youth and adults using the Gallup World Poll data [27].

Two other studies assessed the role that social support plays in the association between food insecurity and mental health, both using Gallup World Poll data. Frongillo et al. [15] conducted their analyses on a global level while Na et al. [16] assessed this association only among adults in sub-Saharan African countries. Another difference is that Na et al. [16] studied a range of support factors: social, emotional and received or given (in the form of money or goods) while Frongillo et al. [15] only evaluated emotional social support. Similar to our findings, Na et al. [16] found that social support was associated with better mental health while controlling for hunger. Also similar to our findings, Frongillo et al. [15] reported that emotional social support did not moderate the association between food insecurity and mental health.

However, in contrast to our and Frongillo et al.’s [15] findings, Na et al. [16] found that social and emotional support moderated the associations with poor health and were associated with more positive health experiences. It is important to note that in addition to the different study populations across the studies, there is a difference in the measure of social support that was adopted. Na et al. [16] measure of social support asked “If you were in trouble, do you have relatives or friends you can count on to help you whenever you need them, or not?” [16]. This indicates that their question captures social support that can include alleviating mental health complaints associated with hunger [16]; our measures and the measures used by Frongillo et al. [15] were more inclined towards emotional support – without the aspect of easing adverse mental health complaints due to hunger. These findings highlight the importance of comparing social support measures across different studies, populations and age groups as they have different associations with hunger, social support and mental health.

As such, our study adds to the literature by investigating a comprehensive set of support factors that surround youth (peers, family, teachers, school climate and neighborhood features) and finds that despite controlling for all these factors; hunger was still associated with lower mental health scores –in-line with the general benefits model [13, 14]. Among our sample of youth in Canada, social support in the form of emotional support did not overpower the negative association between hunger and social support.

Additionally, an important finding from our research is that: although all support factors were associated with positive mental health – there are differences across gender. Females and males perceive [28] and cope with stress differently ([29], justifying our gender-specific analysis and our results showing gender differences. We found that females had lower odds of reporting middle/high support from teachers, school climate and neighborhood features than males. There is controversy on whether there are gender differences in teacher support among youth [30]. As for associations with mental health, among females youth: family, teacher and neighborhood support had higher odds of medium/high mental health relative to males. Whether these findings indicate that these support factors are associated with mental health among females relative to males, or whether these results are due to females and males perceiving support and hunger (as a stressor) differently is an avenue for future research to investigate.

### Recommendations

Our results emphasize the importance of the social environment in its association with positive mental health among youth; this is especially prominent among youth undergoing adversity such as going to bed without supper/dinner because there is not enough food at home. Social support has a relationship with a higher mental health score while controlling for hunger status; some examples include enhancing: parent-child communication [31], the quality of teacher-child interaction [32] and the school’s sense of community and acceptance of diversity [33]. However, hunger due to a lack of food at home is indicative of neglect or social inequality, and policy implementations in Canadian provinces that have been associated with changes in the prevalence of food insecurity should be used to inform other provincial and federal level policies ([34, 35] V [36].).

### Strengths and limitations

This study has the following strengths. We used a representative sample of youth across Canadian provinces and territories making our results generalizable. We also measured five different factors of social support (individually and collectively) to present a comprehensive investigation of the association between the direct social environment with hunger and mental health among youth. Additionally, we conducted gender-specific analysis to account for gender differences in: hunger’s association with mental health, perceived social support and reports of social relationships [10, 14]. Furthermore, we investigated each support factor’s moderating association with hunger via interaction terms; however, it is likely that more than one support factor occurs at the same time with hunger. To keep our analysis and interpretation parsimonious we conducted a two-way interaction; however, it cannot be discounted that evaluating for the – collective – association of the support factors is very likely to have a synergistic effect on mental health.

Despite its strengths, this study is not without limitations. We used measures of perceived support which are subjective measures; yet, this does not discount the directionality of the associations that were identified. Additionally, the cross-sectional design did not allow us to investigate early-life experiences with hunger and their cumulative or longitudinal associations with adolescent mental health.

## Conclusions

Our study sought to identify whether social support plays a role in the association between hunger and mental health among youth among a probability sample of Canadian youth. We find that youth who experience hunger have less perceived support than youth who never experience hunger. This is of importance since hunger is associated with poorer mental health while social support is a protective factor for mental health. Our findings also show that although emotional social support is associated with a higher mental health score when adjusting for hunger status; this type of social support does not obscure the negative association that hunger has on mental health among youth in Canada. We also found that social support and hunger are associated with mental health differently across the genders, prompting future research to continue to stratify analyses by gender for gender-specific results and recommendations.

## Supplementary Information

**Additional file 1: Supplemental Table 1**. Results of the ordinal regression models that regressed mental health (low versus medium/high) on hunger, social support factors and control variables, among youth in Canada in gender-specific analyses. The point estimates are adjusted odds ratios and the intervals show 95% confidence intervals

## Data Availability

The data that support the findings of this study are available from the Public Health Agency of Canada but restrictions apply to the availability of these data, which were used under license for the current study, and so are not publicly available.

## Abbreviations

C.I.: Confidence intervals
HBSC: Health behaviour in school-aged children study
OR: Odds ratio
SES: Socio-economic status
WHO: World health Organization
