# Supplementary material for: Does social support moderate the association between hunger and mental health in youth? A gender-specific investigation from the Canadian Health Behaviour in School-aged Children study
Source: Nutr J. 2020 Dec 5;19:134. doi: 10.1186/s12937-020-00648-3 (PMC7719255; doi:10.1186/s12937-020-00648-3)
Supplement: Supplementary file 1 — Additional file 1: Supplemental Table 1. Results of the ordinal regression models that regressed mental health (low versus medium/high) on hunger, social support factors and control variables, among youth in Canada in gender-specific analyses. The point estimates are adjusted odds ratios and the intervals show 95% confidence intervals [file 12937_2020_648_MOESM1_ESM.docx]

Supplemental Table 1: Results of the ordinal regression models that regressed mental health (low versus medium/high) on hunger, social support factors and control variables, among youth in Canada in gender-specific analyses. The point estimates are adjusted odds ratios and the intervals show 95% confidence intervals.

|  | Hunger | Friend support | Family support | Teacher support | School climate | Neighborhood support | All support factors |
| --- | --- | --- | --- | --- | --- | --- | --- |
|  | Females | | | | | | |
|  | Model 1 | Model 2 | Model 3 | Model 4 | Model 5 | Model 6 | Model 7 |
| Hunger status | |  |  |  |  |  |  |
| Never hungry (Reference) | | |  |  |  |  |  |
| Ever hungry | 0.424*** | 0.422*** | 0.546*** | 0.521*** | 0.507*** | 0.507*** | 0.641*** |
|  | (0.355 - 0.505) | (0.350 - 0.508) | (0.455 - 0.654) | (0.436 - 0.624) | (0.423 - 0.608) | (0.424 - 0.608) | (0.529 - 0.775) |
| Friend support | |  |  |  |  |  |  |
| Low (Reference) | |  |  |  |  |  |  |
| Medium | - | 1.310*** | - | - | - | - | 0.905 |
|  | - | (1.139 - 1.508) | - | - | - | - | (0.773 - 1.060) |
| High | - | 2.820*** | - | - | - | - | 1.426*** |
|  | - | (2.463 - 3.230) | - | - | - | - | (1.196 - 1.699) |
| Family support | |  |  |  |  |  |  |
| Low (Reference) | |  |  |  |  |  |  |
| Medium | - | - | 1.817*** | - | - | - | 1.521*** |
|  | - | - | (1.552 - 2.126) | - | - | - | (1.291 - 1.792) |
| High | - | - | 4.634*** | - | - | - | 2.695*** |
|  | - | - | (4.007 - 5.360) | - | - | - | (2.290 - 3.171) |
| Teacher support | |  |  |  |  |  |  |
| Low (Reference) | |  |  |  |  |  |  |
| Medium | - | - | - | 2.272*** | - | - | 1.394** |
|  | - | - | - | (1.931 - 2.674) | - | - | (1.144 - 1.699) |
| High | - | - | - | 4.614*** | - | - | 1.513*** |
|  | - | - | - | (3.763 - 5.656) | - | - | (1.218 - 1.879) |
| School climate | |  |  |  |  |  |  |
| Low (Reference) | |  |  |  |  |  |  |
| Medium | - | - | - | - | 2.683*** | - | 2.030*** |
|  | - | - | - | - | (2.255 - 3.191) | - | (1.699 - 2.426) |
| High | - | - | - | - | 6.034*** | - | 3.148*** |
|  | - | - | - | - | (4.943 - 7.367) | - | (2.616 - 3.788) |
| Neighborhood support | |  |  |  |  |  |  |
| Low (Reference) | |  |  |  |  |  |  |
| Medium | - | - | - | - | - | 1.826*** | 1.390*** |
|  | - | - | - | - | - | (1.577 - 2.114) | (1.176 - 1.643) |
| High | - | - | - | - | - | 3.365*** | 2.010*** |
|  | - | - | - | - | - | (2.858 - 3.962) | (1.692 - 2.387) |
|  | Males | | | | | | |
|  | Model 8 | Model 9 | Model 10 | Model 11 | Model 12 | Model 13 | Model 14 |
| Hunger status | |  |  |  |  |  |  |
| Never hungry (Reference) | | |  |  |  |  |  |
| Ever hungry | 0.568*** | 0.574*** | 0.671*** | 0.637*** | 0.653*** | 0.632*** | 0.777* |
|  | (0.478 - 0.676) | (0.481 - 0.686) | (0.558 - 0.808) | (0.538 - 0.753) | (0.548 - 0.778) | (0.525 - 0.762) | (0.641 - 0.942) |
| Friend support | |  |  |  |  |  |  |
| Low (Reference) | |  |  |  |  |  |  |
| Medium | - | 1.443*** | - | - | - | - | 1.064 |
|  | - | (1.237 - 1.683) | - | - | - | - | (0.886 - 1.279) |
| High | - | 2.918*** | - | - | - | - | 1.550*** |
|  | - | (2.456 - 3.467) | - | - | - | - | (1.259 - 1.908) |
| Family support | |  |  |  |  |  |  |
| Low (Reference) | |  |  |  |  |  |  |
| Medium | - | - | 1.455*** | - | - | - | 1.318*** |
|  | - | - | (1.254 - 1.689) | - | - | - | (1.123 - 1.547) |
| High | - | - | 3.641*** | - | - | - | 2.351*** |
|  | - | - | (3.052 - 4.344) | - | - | - | (1.954 - 2.828) |
| Teacher support | |  |  |  |  |  |  |
| Low (Reference) | |  |  |  |  |  |  |
| Medium | - | - | - | 1.910*** | - | - | 1.376** |
|  | - | - | - | (1.622 - 2.249) | - | - | (1.137 - 1.665) |
| High | - | - | - | 4.417*** | - | - | 1.902*** |
|  | - | - | - | (3.669 - 5.317) | - | - | (1.481 - 2.443) |
| School climate | |  |  |  |  |  |  |
| Low (Reference) | |  |  |  |  |  |  |
| Medium | - | - | - | - | 1.851*** | - | 1.438*** |
|  | - | - | - | - | (1.613 - 2.125) | - | (1.211 - 1.707) |
| High | - | - | - | - | 4.425*** | - | 2.220*** |
|  | - | - | - | - | (3.766 - 5.199) | - | (1.803 - 2.732) |
| Neighborhood support | |  |  |  |  |  |  |
| Low (Reference) | |  |  |  |  |  |  |
| Medium | - | - | - | - | - | 1.770*** | 1.408*** |
|  | - | - | - | - | - | (1.503 - 2.084) | (1.179 - 1.681) |
| High | - | - | - | - | - | 3.581*** | 2.239*** |
|  | - | - | - | - | - | (3.027 - 4.237) | (1.857 - 2.701) |
| *** p<0.001, ** p<0.01, * p<0.05 | | | | | | | |
| Control variables (not shown): grade, race, socioeconomic status, urban status | | | | | | | |
